# Analysis of Cases with Cerebrospinal Fluid Characteristics Similar to Tuberculous Meningitis

**DOI:** 10.1155/2022/9692804

**Published:** 2022-12-31

**Authors:** Duanhua Cao, Tingting Wang, Yali Wang, Jingzhe Han

**Affiliations:** Department of Neurology, Harrison International Peace Hospital, Hengshui, 050000 Hebei, China

## Abstract

**Purpose:**

The diagnosis of tuberculous meningitis (TBM) is difficult and relies on the patient's clinical presentation and initial cerebrospinal fluid testing. Treatment outcomes for some patients with early consideration of TBM meningitis are often poor. *Patients and Methods*. In this study, we retrospectively analyzed 24 non-TBM patients whose early changes of cerebrospinal fluid were similar to those of TBM through the second-generation cerebrospinal fluid sequencing technology.

**Results:**

All patients included in this study had an acute onset, including 5 patients with a history of upper respiratory tract infection, 9 patients with fever, 6 patients with headache, 5 patients with psychiatric symptoms, 6 patients with cognitive impairment, 9 patients with signs of meningeal irritation, and 6 patients with seizures. Sixteen patients presented with altered content and level of consciousness during their admission. The leukocyte counts (median, 124.0 × 10^6^/L) and total protein concentrations (median, 1300 mg/L) were higher than normal reference values in all patients, whereas glucose (median, 1.345 mmol/L) and chloride concentration values (average, 111.7 ± 5.2 mmol/L) were lower than normal reference values. The patients included 2 cases of Liszt's meningitis, 2 cases of Brucella infection in the CNS, 4 cases of Varicella zoster virus encephalitis, 2 cases of human herpes simplex virus type 1, 2 cases of lupus encephalopathy, 2 cases of anti-NMDAR receptor encephalitis, 2 cases of meningeal carcinomatosis, 5 cases of cryptococcal meningitis, 2 cases of CNS sarcoidosis, and a case of invasive Rhizopus oryzae infection. All patients were tested for NGS in cerebrospinal fluid. Eight patients were diagnosed with anti-NMDAR encephalitis, meningeal carcinomatosis, lupus encephalopathy, and CNS sarcoidosis. Nine patients experienced death; 15 patients had a good prognosis and left no significant sequelae.

**Conclusion:**

The analysis of patients with TBM-like cerebrospinal fluid changes will help improve the diagnostic accuracy of the disease and reduce misdiagnosis and underdiagnosis.

## 1. Introduction

Tuberculous meningitis (TBM) is the most common central nervous system (CNS) tuberculosis with high morbidity and mortality, which is still difficult to diagnose because of its lack of specificity in clinical manifestations and low sensitivity in laboratory examination. Cerebrospinal fluid (CSF) examination is the key to the diagnosis of most patients with suspected intracranial infection. The CSF of TBM is characterized by an increase in protein (>450 mg/L), a decrease in glucose (<2.50 mmol/L), and an increase in the number of leukocyte (>8 × 10^6^/L). It is generally believed that the decrease of chloride content in CSF is related to TBM [1]. However, with the development of mNGS technology for CSF pathogens, the final diagnosis was not TBM, which was found to be consistent with the clinical features of encephalitis or meningitis, and the CSF changes were consistent with classic “tuberculous meningitis”-like changes. Therefore, it is currently believed that the classic tuberculous cerebrospinal fluid changes, such as elevated cerebrospinal fluid protein, decreased sugar, and chloride, are of limited value in the early diagnosis of TBM, and the false-positive rate is relatively high. Since it was first reported in 2014, NGS has been used to detect pathogens in the cerebrospinal fluid and can be used rapidly and effectively in the diagnosis of CNS infectious diseases. CNS can quickly and efficiently diagnose infectious diseases and has been used to detect pathogens in cerebrospinal fluid since NGS was first reported in 2014 [2]. In this study, the cerebrospinal fluid mNCS technique was used to detect and analyze the clinical features of early cerebrospinal fluid changes in 24 patients with non-TBM with classical TBM-like features. The study will improve clinicians' understanding of patients with this TBM-like disorder and reduce misdiagnosis and underdiagnosis.

## 2. Material and Methods

### 2.1. Study Subjects

A total of 24 patients with non-TBM who were admitted to our Department of Neurology from January 2017 to June 2021 and diagnosed with early cerebrospinal fluid changes consistent with the characteristics of TBM were selected. This study was approved by the Ethics Committee of Hengshui People's Hospital. The patients and their families were informed of the contents of the study and signed the informed consent form.

Inclusion criteria are as follows: (1) clinically suspected encephalitis or meningitis, early CSF changes are similar to TBM (increased CSF protein (>450 mg/L), decreased glucose (<2.50 mmol/L), and chloride (<120 mmol/L) and (2) no evidence of tuberculosis infection in the CNS was found in clinical and laboratory examinations, and the final diagnosis was non-TBM.

Exclusion criteria are as follows: (1) CNS tumor and space-occupying disease, (2) contraindication of lumbar puncture or inability to obtain cerebrospinal fluid, (3) refuse to test for cerebrospinal fluid pathogen mNGS, and (4) incomplete clinical data and irregular case records.

### 2.2. Research Methodology

Retrospective analysis of the included cases, the basic information, prodromal symptoms (upper respiratory tract infection), other clinical symptoms such as memory decline, mental symptoms, cognitive impairment, level of consciousness and content changes, meningeal irritation, seizures, history, EEG, treatment, imaging including head magnetic resonance (MRI) and lung CT, and examination data (including haematoma series, erythrocyte sedimentation rate, blood rheumatic series, cranial pressure, serum and cerebrospinal fluid autoantibodies and related routine tests, and results of cerebrospinal fluid pathogen mNGS) were recorded.

Patients with upper respiratory tract infection, psychiatric symptoms (delirium and confusion), and cognitive functions such as calculation, comprehension, and orientation were detected by MMSE scale and MoCA scale (Mini-Mental State Examination (MMSE) < 24 is abnormal; Montreal Cognitive Assessment (MoCA) < 26 is abnormal). The level or content of consciousness was detected by GCS score; meningeal stimulation sign was considered abnormal, seizure occurrence was considered abnormal, EEG suggesting encephalitis was considered abnormal, and blood sedimentation > 20 was considered abnormal (normal < 20). Rheumatoid factor > 20 IU/mL is abnormal (normal, 0-20 IU/mL), cranial pressure > 180 mm H_2_O is abnormal, and antibodies found in the cerebrospinal fluid of anti-NMDA receptor encephalitis are positive; cerebrospinal fluid second-generation sequencing found pathogenic pathogens is clinically significant.

### 2.3. Immunofluorescence

Antibodies against autoimmune encephalitis were detected by indirect immunofluorescence method based on tissue cells, anti-globulin antibodies were labelled with fluorescein, and fluorescent-labelled anti-globulin antibodies interact with bound antibodies to infer the presence of antigens or antibodies. Under fluorescence microscope observation, if granular fluorescent particles were found in the cell membrane and cytoplasm, they were positive.

### 2.4. Second-Generation Sequencing

The following is the second-generation sequencing of cerebrospinal fluid: (1) sample collection: 1-2 mL cerebrospinal fluid was obtained by a lumbar puncture and then dropped into a test tube (Sarstedt, Germany) and stored in 80°C refrigerators within 30 minutes for second-generation sequencing. (2) Sample extraction and quality control: the DNA of cerebrospinal fluid was extracted by a microsample genomic DNA extraction box (DP316, Tiangen Biochemical Technology (Beijing) limited company); the DNA was broken down to 200-300 bp fragments by DNA cutting ultrasonic cracker (Q800R2, Qsonica, USA), 2100 biological analyzer (Agilent Company, USA) quality control fragment size, and quantitative PCR (Thermo Fisher Company, USA) quality control DNA library concentration. (3) Library construction: after the DNA fragments were repaired and A added, the splice sequences were added at both ends and then cycled to form single-stranded circular DNA; rolling-loop amplification increased the single-stranded circular DNA by 2-3 number sets and obtained the DNA nanospheres. (4) Sequencing: the DNA nanoparticles were loaded onto the sequencing chip and sequenced DNA nanospheres using the BGISEQ-50 sequencing platform (Medical Laboratory of Tianjin Huada Gene Technology Co., Ltd.). (5) Data analysis and quality control: after the sequence data is disassembled, the reads with low quality, low complexity, and sequence length less than 35 bp are eliminated; the high-quality sequence data are obtained and compared with the BWA human genome database. Then, the interference of human genome sequence information was eliminated. The remaining data are compared with microbial databases to enable preliminary identification of bacteria, viruses, fungi, and pathogenic microorganisms. The main outcome measures were pathogens associated with infectious diseases of the CNS. Nonbackground sequences detected by the second-generation sequencing of cerebrospinal fluid were used to identify the pathogens with sequence number > 2.

### 2.5. Statistical Analysis

All statistical analyses of the data were calculated using SPSS 25.0. Also, the mean ± standard deviation was used to express the continuous data that conformed to a normal distribution; the median, 25%, and 75% values were used to express the continuous data that did not conform to a normal distribution.

## 3. Results

### 3.1. Clinical Features

A total of 24 patients with non-TBM who were admitted to our Department of Neurology from January 2017 to June 2021 and diagnosed with early cerebrospinal fluid changes consistent with the characteristics of TBM were selected. The non-TBM patients included 12 males and 12 females, aged 21-78 years old, with an average of 48.13 ± 14.6 years old. The patients included 2 cases of Liszt's meningitis, 2 cases of Brucella infection in the CNS, 4 cases of Varicella zoster virus encephalitis, 2 cases of human herpes simplex virus type 1, 2 cases of lupus encephalopathy, 2 cases of anti-NMDAR receptor encephalitis, 2 cases of meningeal carcinomatosis, 5 cases of cryptococcal meningitis, 2 cases of CNS sarcoidosis, and a case of invasive Rhizopus oryzae infection (Table 1).

All patients in the group had acute onset. There were 5 cases of upper respiratory tract infection, fever (9 cases), headache (6 cases), mental symptom (5 cases), cognitive disorder (6 cases), meningeal irritation (9 cases), and epilepsy (6 cases). The changes of consciousness content and (or) consciousness level occurred in 16 patients during admission, which was as follows: confusion (8 cases), apathy (1 case), delirium (2 cases), deep coma (1 case), light coma (2 cases), and somnolence (2 cases); 11 cases were transferred to ICU during hospitalization. More details are shown in Table 1.

### 3.2. Laboratory Examination

For cerebrospinal fluid, all patients underwent lumbar puncture and cerebrospinal fluid examination in our hospital. The leukocyte count was increased (>8 × 10^6^/L), and the median value is 124.0 × 10^6^/L (89.0 × 10^6^/L and 490.5 × 10^6^/L); total protein increased (>450 mg/L), and the median value is 1300 mg/L (1009.5 mg/L and 2314.5 mg/L); glucose decreased (<3.66 mmol/L), and the median value is 1.345 mmol/L (1.045 mmol/L and 2.478 mmol/L); and chloride decreased (<120 mmol/L), and the average value is 111.7 ± 5.2 mmol/L. More detailed results of the patients are shown in Table 2.

All patients were tested for NGS in cerebrospinal fluid, and the pathogen types and copy sequences are shown in Table 2. Eight patients were diagnosed with anti-NMDAR encephalitis, meningeal carcinomatosis, lupus encephalopathy, and CNS sarcoidosis. Anti-NMDAR encephalitis and lupus encephalopathy were confirmed by antibody test results. Meningeal carcinomatosis was confirmed by cerebrospinal fluid cytology, and the CNS sarcoidosis met the clinical diagnostic criteria. Video EEG results show that except 6 who were examined during hospitalization, other patients showed an extensive moderate or above abnormality.

### 3.3. Imageology

**Figure 1:**
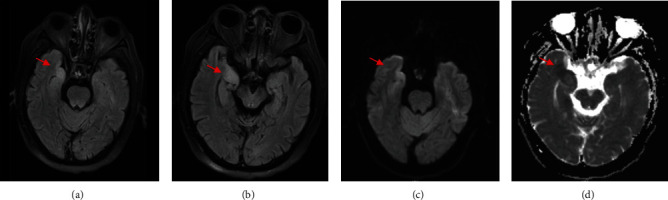
MRI findings in a patient with herpes simplex virus encephalitis. (a) Flair sequence shows high signal in the right temporal lobe. (b) Flair sequence showing high signal in the right hippocampus. (c) DWI sequence showing high signal in the right temporal lobe. (d) Right temporal lobe low signal. The red arrow indicates the lesion area.

All the patients were examined by cranial MRI. A total of 10 patients had abnormal findings on cranial MRI, including 2 cases of anti-NMDA encephalitis. The two patients showed abnormal signals in the unilateral temporal lobe, hippocampus, and another limbic system. One case of meningeal carcinomatosis showed enhancement of pia mater by enhanced MRI. Two cases of meningococcal disease showed meningeal enhancement and basal ganglia infarction. Two cases of VZV encephalitis showed diffuse brain parenchymal lesion accompanied by cranial nerve enhancement. Two cases of HSV1 encephalitis showed abnormal signals in the right hippocampus and insula. One case of Liszt's encephalitis suggested brainstem damage (Figure 1).

### 3.4. Treatment and Prognosis

All patients were treated according to diagnosis, and partial patients were treated with immunosuppressive therapy (glucocorticoid and intravenous immunoglobulin 0.4 g·kg/d treatment for 5 days). None of the patients had any adverse reactions. Six-month follow-up showed that 9 patients died (5 cases of cryptococcal meningitis, 2 cases of meningeal carcinomatosis, 1 case of Rhizopus oryzae infection, and 1 case of severe VZV encephalitis). 15 cases had a good prognosis without obvious sequelae.

## 4. Discussion

The diagnosis of TBM is difficult and mainly depends on the clinical manifestations and initial cerebrospinal fluid characteristics. The result of staining showed that acid-fast bacilli have important value for the diagnosis of TBM. However, the quantity of CSFs, the timeliness of sending samples for analysis, and the experience of the inspectors resulted in a low positive rate of acid-fast staining [3]. Bacterial culture also has the disadvantages of long cycles and low sensitivity. Thus, some new methods for TBM diagnosis have been adopted in recent years, such as ELISA/TBM DNA level detection. However, finding definitive evidence of CNS tuberculosis infection remains a major challenge, regardless of the testing method used. Therefore, most guidelines and consensus clearly state that once TBM is clinically suspected, the evidence must be evaluated quickly and thoroughly and a decision was made as soon as possible. This is because early diagnosis and treatment are the most important factors affecting the prognosis of patients with TBM. Therefore, the diagnosis of TBM requires early recognition of changes in the cerebrospinal fluid. Studies have shown that although the cerebrospinal fluid in some diseases has a similar profile to that of TBM and is treated early with antituberculosis, some cases are still found to be nontuberculous infections. With the development and application of cerebrospinal fluid mNGS technology, some patients were found to have early cerebrospinal fluid changes similar to TBM but were eventually diagnosed with non-TBM infections. It is feasible for early treatment with anti-tuberculosis therapy by TBM which is clinically suspected. However, the diagnosis of TBM is inaccurate when TBM-like cerebrospinal fluid changes are present. The differential diagnosis requires full consideration of the patient's clinical features and exclusion of the possibility of cerebrospinal fluid changes caused by tuberculous meningitis disease. An accurate differential diagnosis can not only reduce the occurrence of adverse reactions in patients due to the irrational use of antituberculosis drugs but also help to provide early treatment for patients, reduce the false-positive rate of TBM, and improve the prognosis. In this study, the clinical features of the patients who were diagnosed as non-TBM were analyzed retrospectively, and the clinical features were summarized to serve the clinic.

Although the early cerebrospinal fluid changes in all patients in this study were similar to those seen at the onset of tuberculous meningitis, there still had unique clinical features. First, there are certain specific clinical manifestations, such as the long duration of clinical symptoms (all > 6d) and the presence of focal signs in all patients in this study. The prodromal symptoms in some adult TBM patients may manifest as subacute dementia, with patients often experiencing a slow progression over months or even years. In contrast, the late stage of TBM may present as a rapidly progressive meningitis syndrome and is difficult to distinguish from other intracranial infections with meningitis. In this study, all patients had an acute onset of meningitis with acute meningitis-like changes such as fever, headache, acute impaired consciousness, cognitive impairment, seizures, and psychiatric symptoms. The patients did not have the slowly progressive clinical manifestations typical of TBM, and none of them had focal localization signs. All patients in this study had an acute onset, short duration, and strong inflammatory response. Second, most TBM is a chronic infectious disease, and the most common imaging findings on MRI are basement membrane enhancement, hydrocephalus, and infarct occurrence in the supratentorial brain parenchyma and brainstem [4]. MRI and diffusion-weighted imaging can help detect early infarction in TBM. Magnetic resonance angiography reveals vascular changes in patients and is commonly seen in the distal internal carotid artery and the proximal middle and anterior cerebral arteries [5]. In this study, MRI+MRA and enhanced MRI of the head were performed in all patients. Although some patients showed meningeal enhancement, none of them showed basement membrane enhancement-like features similar to chronic infection, and MRA did not show clear evidence of infection-induced large vessel stenosis. None of the above patients are consistent with the imaging features of TBM. Although basal ganglia infarction was seen in some cryptococcal meningitis found in this study, no definite vascular stenosis was found in the large vessels. Basal ganglia infarction was considered to be associated with microvasculitis caused by cryptococcal infection. This is crucial for the clinical recognition of TBM and cryptococcal infection [6]. Again, early cerebrospinal fluid changes are highly suggestive of TBM, such as (i) elevated cell counts (100-500/*μ*L), mainly lymphocytes and possibly neutrophils in the early stages of the disease; (ii) elevated protein levels (up to 1000-5000 mg/L); and (iii) decreased glucose levels (<2.5 mol/L) or cerebrospinal fluid/plasma ratios < 0.5 [7–9]. In the present study, the early cerebrospinal fluid leukocyte count, protein level, and glucose level of all enrolled patients were similar to the cerebrospinal fluid changes in TBM. Therefore, early reliance on cerebrospinal fluid changes alone to rule out TBM disease is difficult and requires a combination of patient clinical features and various ancillary tests to achieve the goal [10].

The normal cerebrospinal fluid cell count usually does not exceed 5 cells/*μ*L and is dominated by monocytes and lymphocytes. CNS infection, tumors, trauma, brain parenchymal hemorrhage, or lumbar puncture can cause an increase in the cerebrospinal fluid cell count. Therefore, an increase in cerebrospinal fluid cell count is not typical for the disease. Cellular responses in the cerebrospinal fluid have been classified into 3 types: infectious and autoimmune inflammatory diseases, nonspecific stimulatory processes, and neoplastic changes [11]. Most uncomplicated viral meningitis ends the granulocyte cycle in a matter of hours or days. Therefore, the first cerebrospinal fluid examination in uncomplicated viral meningitis will show a lymphocyte-dominant appearance and a milder occurrence of blood-cerebrospinal fluid barrier dysfunction compared to bacterial meningitis. In the present study, the altered cerebrospinal fluid cell count in viral meningitis was generally consistent with these features. Meningeal carcinomatosis was similarly accompanied by an increase in reactive inflammatory lymphocytes. Therefore, patients with meningeal carcinomatosis can also present with elevated leukocytes, mainly lymphocytes [12]. The development of autoimmune encephalitis shows activation of the central nervous immune system. This process is usually combined with abnormalities of the cerebrospinal fluid, such as cerebrospinal fluid leukocytosis (>5 × 10^6^/L), cerebrospinal fluid showing lymphocytic inflammation, and positive cerebrospinal fluid oligoclonal zone banding. However, in most cases, the leukocyte count does not exceed 100 × 10^6^/L. In this study, the cerebrospinal fluid changes in NMDAR receptor encephalitis, lupus encephalopathy, and CNS nodal disease resembled these features. Therefore, elevated cerebrospinal fluid leukocytes are only indicative of “inflammatory” response; however, the exact type of inflammatory response needs to be further analyzed.

The normal reference range of cerebrospinal fluid proteins varies depending on age and source of specimen. Increased protein levels in cerebrospinal fluid usually indicate the occurrence of increased permeability of the blood-brain barrier, decreased absorption of arachnoid granules, obstruction of cerebrospinal fluid circulation, and increased synthesis of intrathecal globulins [13]. These conditions are usually seen in infectious diseases of the central nervous system, such as septic meningitis, tuberculous meningitis, neurosyphilis, viral encephalitis, and fungal infections; they are also seen in noninflammatory diseases, occupying central tumors, and cerebral hemorrhage. All patients in this study had cerebrospinal fluid protein levels above the normal reference range (>450 mg/L). We believe that this may be closely related to the permeability of the blood-brain barrier. However, for autoimmune-related cases, the occurrence of increased cerebrospinal fluid protein levels may also be related to intrathecal synthesis. However, none of the patients included in this study were analyzed for cerebrospinal fluid-serum-albumin ratio (QAlb) and immunoglobulins.

Decreased cerebrospinal fluid glucose levels are most often seen in septic, tuberculous, and fungal meningitis. CNS metabolites also affect the functioning of the blood-brain barrier (BBB) for sugar carriage, causing a decrease in the amount of glucose entering the CSF in the blood. Studies have shown that glucose levels in the blood rise rapidly after intravenous administration of hypertonic glucose in patients with tuberculous and fungal meningitis but rise insignificantly in the CSF. Therefore, the decrease of glucose in CSF can be an important indicator of bacterial or fungal infection in the CNS [14]. Reduced cerebrospinal fluid glucose is also seen in meningeal carcinomatosis and intracranial parasitic infections. In meningeal carcinomatosis, metabolically active cancer cells can contribute to the rapid enzymolysis of glucose in the CSF and prevent glucose infiltration into the BBB. Glucose levels in the CSF can fall to zero in meningeal carcinomatosis, which can be important diagnostic evidence [15]. Among the brain, parasitic diseases, such as cerebral cysticercosis, schistosomiasis, pulmonary schistosomiasis, and toxoplasmosis, can contribute to the decrease in CSF glucose levels. Some of the disease spectrum cerebrospinal fluid glucose levels in the patients included in this study were generally consistent with changes in cerebrospinal fluid glucose levels. However, a decrease in glucose levels was observed in this study regarding viruses, autoimmune encephalitis, and neurological nodal disease. This is inconsistent with other studies reported. We believe that it may be related to several reasons, such as increased permeability of the blood-brain barrier, which leads to increased glucose leakage from the CSF; short-term or persistent vasoconstriction or narrowing caused by local cerebrovascular inflammation; and abnormal cerebral hemodynamics, which causes a decrease in glucose transport into the CSF. However, the exact cause of glucose abnormalities still needs further destudies to confirm.

Normal cerebrospinal fluid has a higher chloride content than plasma, mainly due to the presence of Donnan balance in cerebrospinal fluid. Therefore, the amount of chloride in the cerebrospinal fluid varies with changes in plasma chloride levels [16]. When the cerebrospinal fluid osmolarity is maintained in equilibrium, an increase in protein content causes a decrease in chloride. Decreases in chloride levels are seen in various central nervous system infections, most notably in tuberculous meningitis disease. In cases of hypochlorhydria (vomiting, dehydration, etc.), cerebrospinal fluid chloride is also reduced. Therefore, changes in cerebrospinal fluid chloride levels need to be analyzed with full consideration of blood chloride concentration levels. In the present study, a decrease in cerebrospinal fluid chloride levels occurred in all patients. The main reason for this may be related to an increase in cerebrospinal fluid protein levels and, in part, to a decrease in blood chloride levels. However, all of the included patients showed a mild decrease in cerebrospinal fluid chloride levels, and the decrease was significantly lower than the TBM reference value. The specific mechanisms involved in the above results still need to be further investigated.

## 5. Conclusion

This study suggests that differences in cerebrospinal fluid protein levels and chloride levels may be characteristic indicators for the differential diagnosis of TBM and similar diseases, but further studies are needed. Since this study is a single-centre study and there is no control group, the findings of this study are for clinical reference only. More similar studies are needed in the future. Therefore, in the event of TBM-like cerebrospinal fluid changes, we recommend a comprehensive analysis of the patient's clinical features, cerebrospinal fluid changes, and imaging features. Considering the existence of a disease spectrum that mimics TBM, a good differential diagnosis should be made as a way to achieve an accurate diagnosis of TBM. At the same time, reducing the number of missed diagnoses of TBM-like diseases can achieve the goal of improving patient prognosis.

## Figures and Tables

**Table 1:** Number of patients presenting with symptoms.

General information	Front wheel drive	Fever	Headache	Mental symptoms	Cognitive impairment	Disturbance of consciousness	Meningeal irritation	Epileptic seizure	ICU
Total (24) (%)	5 (20.8%)	10 (41.7%)	8 (33.3%)	5 (20.8%)	6 (25%)	16 (66.7%)	9 (37.5%)	6 (25%)	11 (45.8%)
Liszt's meningitis (2)	1	1	1	1	—	1	1	1	1
Brucella (2)	1	1	—	—	—	2	1	—	1
Varicella zoster virus encephalitis (4)	2	2	1	2	2	2	—	2	2
Herpes simplex virus type 1 (2)	—	1	1	—	1	2	—	—	1
Lupus encephalopathy (2)	—	1	1	1	1	1	—	2	1
NMDAR receptor encephalitis (2)	—	1	1	—	1	1	1	—	1
Meningeal carcinomatosis (2)	—	1	—	1	1	2	—	—	1
Hidden meningococcal disease (5)	1	1	1	—	—	3	5	—	2
Central nervous system sarcoidosis (2)	—	—	1	—	—	1	—	1	1
Rhizopus oryzae infection (1)	—	1	1	—	—	1	1	—	—

**Table 2:** The characteristics of cerebrospinal fluid.

No.	Gender (M/F)	Name	Age	Pressure	WBC (×10^6^/L)	Protein (mg/L)	Glucose (mmol/L)	Chloride (mmol/L)	NGS (SN)	Cytology	Antibody detection
1	M	Liszt's	38	300	492	2085	0.6	111.2	122	Mixed cytological reaction	Negative
2	M	Liszt's	42	320	486	1767	0.8	113.4	146	Mixed cytological reaction	Negative
3	M	Brucella	48	402	92	960	3.15	114	1263	The lymphocyte reaction dominant	Negative
4	M	Brucella	66	350	286	1260	1.27	112	867	The lymphocyte reaction dominant	Negative
5	M	HSV3	60	330	567	11368	2.49	105.8	20161	The lymphocyte reaction dominant	Negative
6	M	HSV3	21	180	556	5418	3.52	119.2	11930	The lymphocyte reaction dominant	Negative
7	M	HSV3	72	220	576	6273	2.11	103.2	12360	The lymphocyte reaction dominant	Negative
8	M	HSV3	54	190	579	4321	1.26	110.5	13570	The lymphocyte reaction dominant	Negative
9	M	HSV1	35	110	41	530	1.06	110.6	13174	Lymphocyte	Negative
10	F	HSV1	44	270	102	720	2.08	100.2	20162	Lymphocyte	Negative
11	M	Lupus	52	110	123.0	2391	2.44	118.3	Negative	Lymphocyte	Negative
12	M	Lupus	58	210	88	1369	1.22	106.2	Negative	Lymphocyte	Negative
13	F	NMDA	68	180	56	1210	2.36	116.2	Negative	Lymphocyte	Anti-NMDA
14	F	NMDA	54	200	92	1420	3.22	113.4	Negative	Lymphocyte	Anti-NMDA
15	F	MC	32	180	33	812	4.18	119.0	Negative	Heterocyst	Negative
16	F	MC	38	240	87	1038	1.42	116.5	Negative	Heterocyst	Negative
17	F	Cryptococcus	38	380	80	878	2.41	116.7	3026	The lymphocyte reaction dominant	Negative
18	F	Cryptococcus	32	360	620	1112	1.22	108.3	8324	The lymphocyte reaction dominant	Negative
19	F	Cryptococcus	78	280	170	1230	1.26	106.2	2083	The lymphocyte reaction dominant	Negative
20	F	Cryptococcus	65	220	150	1340	1.02	104.8	8235	The lymphocyte reaction dominant	Negative
21	F	Cryptococcus	44	320	230	1164	1.04	112.2	917	The lymphocyte reaction dominant	Negative
22	F	CNSS	38	260	121	2442	0.42	114.2	Negative	Monocyte cell dominated	Negative
23	F	CNSS	42	220	125	1830	0.68	116.4	Negative	Monocyte cell dominated	Negative
24	M	ROI	36	150	109	1000	5.54	111.2	886	Multinucleate cell dominated	Negative

M: male; F: female; Lupus: lupus encephalopathy; NMDA: NMDAR receptor encephalitis; MC: meningeal carcinomatosis; HMD: hidden meningococcal disease; HSV: herpes simplex virus; CNSS: central nervous system sarcoidosis; ROI: Rhizopus oryzae infection; URI: upper respiratory infection; WBC: white blood cell; SN: sequence number.

## Data Availability

All data generated or analyzed during this study are included in this article.
